# Spinal Epidural Abscess: A Review Highlighting Early Diagnosis and Management

**DOI:** 10.31662/jmaj.2019-0038

**Published:** 2019-10-24

**Authors:** Syuichi Tetsuka, Tomohiro Suzuki, Tomoko Ogawa, Ritsuo Hashimoto, Hiroyuki Kato

**Affiliations:** 1 Department of Neurology, International University of Health and Welfare Hospital, Nasushiobara, Japan

**Keywords:** spinal epidural abscess, diagnostic delay, MRI findings, neurologic deficits

## Abstract

Spinal epidural abscess (SEA) is still an uncommon but devastating infection of the spine. In recent years, a number of reported cases have risen. The most important prognostic factor for a favorable outcome is early diagnosis and appropriate treatment. However, a diagnosis of SEA is often delayed, particularly in the early stages of the disease before patients present with neurological symptoms. With enough knowledge of risk factors, clinical features, and appropriate diagnostic procedures, it may be possible to reduce diagnostic delay in the early stages of the disease. This review focuses on early diagnosis of SEA based on risk factors, presenting symptoms, and characteristic findings on magnetic resonance imaging (MRI), and also discusses the timing of surgical interventions. Traditionally, the symptoms of SEA are characterized by fever, back pain, and neurological symptoms, which are described as a classical triad of symptoms for this type of infection; but this collection of symptoms is seen in only about 10% of cases. However, most patients complain of severe localized lower back pain. Gadolinium-enhanced MRI is the most sensitive, specific, and beneficial imaging modality for establishing a diagnosis of SEA. Patients diagnosed prior to neurological deficits with a known causative microbial organism can be safely treated with antimicrobial therapy alone. However, about 30%–40% of the patients fail in conservative management without surgery. The best management and timing for surgical decompression in patients with or without mild neurological deficits should be established in the near future. Early diagnosis and management, before the occurrence of serious neurological symptoms, are the most important prognostic factors for good outcomes in patients with SEA. We proposed a simple algorithm for early diagnosis of SEA by selecting patients with severe back pain, leading to emergent MRI.

## 1. Introduction

Spinal epidural abscess (SEA) is a rare disease characterized by the accumulation of pus in the epidural space causing compression of the spinal cord and spinal roots. During the 1970s, its incidence was reportedly 0.2 to 2.0 cases per 10,000 hospitalized patients ^(1)^. However, with the increased use of magnetic resonance imaging (MRI), the detection rate is improving, and consequently, the number of reported cases is rising ^(2), (3), (4)^. Several patients with SEA suffer from low back pain and are then referred to a medical institution. The diagnosis of SEA, however, is not often made at the first visit, and the treatment initiation is commonly delayed. Lower-extremity weakness or bladder/rectal dysfunction during follow-up often signifies SEA. If neurological conditions such as sensory disturbance occur, they sometimes persist and cause serious and irreversible sequelae of events. Therefore, an appropriate therapeutic intervention is important immediately after the diagnosis ^(5)^. The rate of initial misdiagnosis has been historically described as high as 75%–89%, which could result in permanent neurological dysfunction, paralysis, and death of patients ^(6), (7)^.

In this review, we aim to improve outcomes for patients with SEA by providing clinicians with a general overview of the disease and understanding of patients’ risk factors, presenting symptoms, and radiological characteristics of SEA on MRI. Furthermore, we proposed a simple algorithm for early diagnosis and discussed the timing of appropriate surgical interventions.

## 2. Anatomy

**Figure 1.**
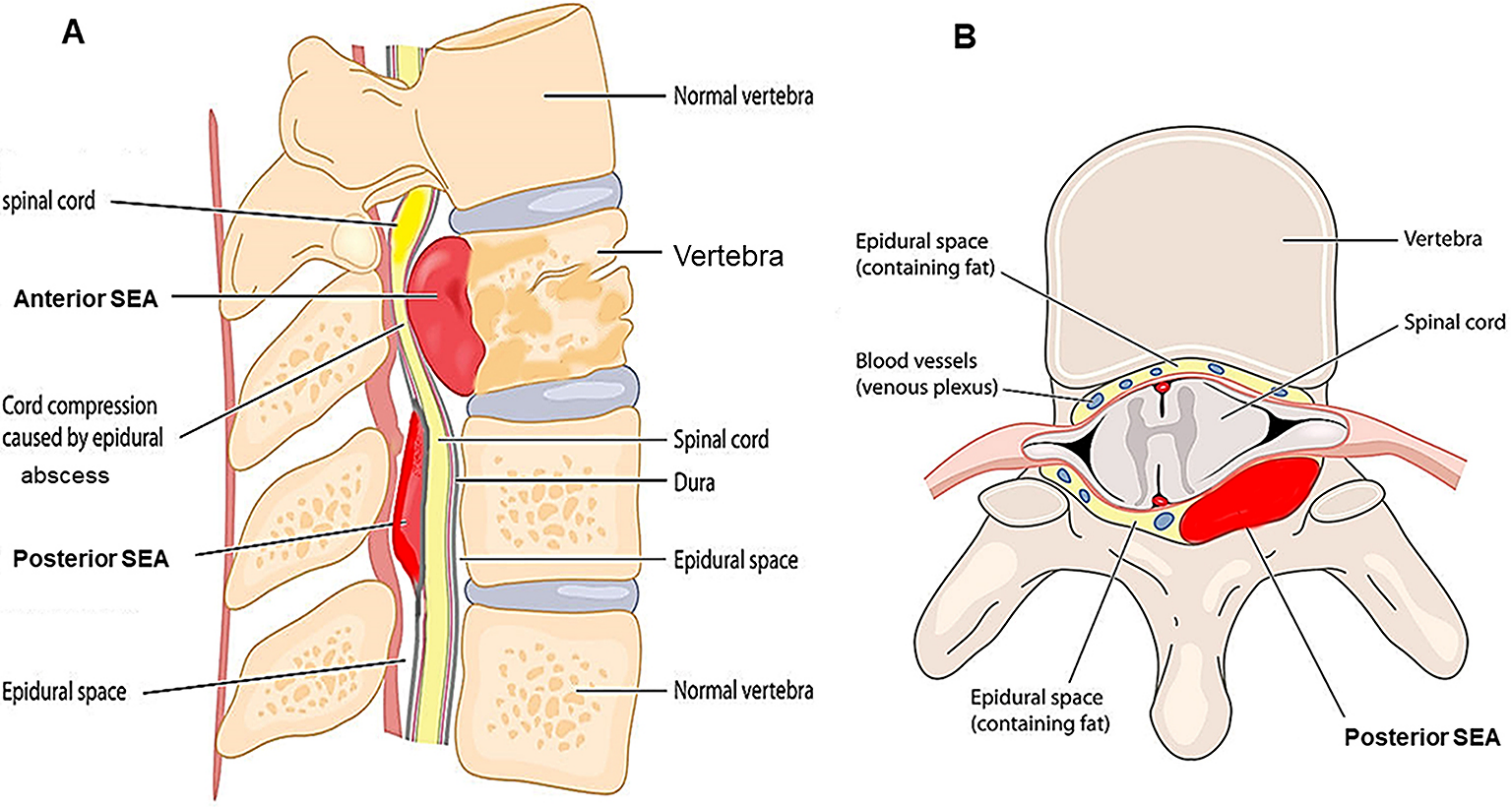
Anatomy of the spine with a spinal epidural abscess. Longitudinal and transverse section of a vertebral body. SEA, spinal epidural abscess.

The clinical features of epidural abscesses are dependent on the anatomy of the spinal canal and dural tube. The epidural space is smaller in the cervical region and larger in the lumbosacral region. The development and localization of epidural abscesses are related to the presence of a real epidural space. Usually, the dura mater adheres superiorly at the foramen magnum and inferiorly at the sacrococcygeal membrane. The anterior epidural space is mostly occupied by the dura, posterior longitudinal ligament, and periosteum of the vertebral body, which are tightly adherent; thus, most SEAs occur posteriorly ^(8)^. The epidural space contains fat, arteries, and venous plexus. SEAs are more common in the thoracolumbar area, where the epidural space is larger and contains more infection-prone fat tissue, and the low pressure of the venous plexus can easily cause reflux from the venous plexus within the abdominal and pelvic cavities at the same regions ^(9), (10)^. As mentioned, a vast number of SEAs are located posteriorly, but most anterior SEAs occur below the level of L1 (Figure 1). When SEAs are caused secondly to pyogenic spondylitis or discitis, they are often located anterior to the dural tube. On the contrary, in cases of hematogenous infection, they are often located posterior to the dural tube. Furthermore, the vertical sheath nature of the epidural space allows the spread of abscesses from the level of origin to multiple levels longitudinally.

## 3. Pathogens and Pathophysiology

Methicillin-sensitive *Staphylococcus aureus* (MSSA) is the most common causative pathogen of SEA, accounting for about two-thirds of cases, followed by other gram-positives, *Escherichia coli*, etc. Recently, the number of methicillin‐resistant *Staphylococcus aureus* (MRSA) has significantly increased ^(11), (12), (13)^. Several other bacteria can also cause such infection. Among aerobic gram-negative bacilli, *E. coli* often causes SEA in patients with urinary tract infection (UTI).

Infection can be due to direct transmission from a nearby infection, hematogenous infection from a local or lymphatic infection, or**iatrogenic inoculation, but some cases show no obvious source of infection, and several are associated with immunodeficiency ^(14), (15)^. Because the extradural space has abundant blood flow, hematogenous infection from UTI or infective endocarditis is common. Several SEAs begin as a focal pyogenic infection involving the vertebral disk or junction between the disk and vertebral body ^(16), (17)^. As the pyogenic inflammation progresses and the abscess extends longitudinally within the epidural space, the spinal cord can be damaged due to the following mechanisms: (i) direct compression, (ii) thrombosis and thrombophlebitis of nearby veins, (iii) interruption of arterial blood supply, and (iv) inflammation caused by bacterial toxins and mediators. In particular, the latter mechanism is hypothesized to begin with* S. aureus* as the causative agent. The pathogenic agents of *S. aureus* are roughly classified into exotoxins, extracellular enzymes, and cell surface substances ^(18)^. Among these, the extracellular enzyme coagulase binds to prothrombin to coagulate plasma. A cell surface clamping factor protein and compact colony-forming active substance cause cell aggregation in the presence of fibrinogen, which is involved in blood coagulation ^(19)^. Histopathologically, as the spinal cord lesion in SEA has a wider intravascular thrombus than mechanical compression alone, bacterial infection considerably results in ischemic changes and neural tissue destruction ^(20)^.

In patients with a history of intravenous drug use, the location of the SEA may be correlated with the site of drug injection ^(12)^. As to the iatrogenic inoculation, 15%–22% of epidural abscesses are reported due to invasive instrumentation, such as spinal surgery ^(21), (22), (23)^. Moreover, the incidence of infection after intraoperative epidural anesthesia is about 1 in 2,000, while the long-term use of an epidural catheter may be associated with an infection rate as high as 4.3% ^(15)^. While the catheter is connected, bacteria can reach the epidural space along the catheter track in injected solutions and by local or hematogenous spread.

## 4. Risk Factors or Predisposing Conditions

Following factors predisposing to SEA are known: diabetes mellitus, intravenous drug and long-term systemic corticosteroid therapy, spinal abnormalities such as recent trauma or surgery, epidural anesthesia, treatment around the spinal cord, and systemic bacterial infection from a local infection and injection. In particular, diabetes mellitus and intravenous drug use are considerably relevant ^(5), (14), (15), (24)^. Local or systemic infections include skin and soft tissue infection, osteomyelitis, UTI, infectious endocarditis, and indwelling vascular access infection. In addition, degenerative disk disease, large osteophytes, and chronically hypertrophied facet joints may be the targets of hematogenous bacterial seeding as local spinal risk factors. But they should not be overlooked ^(2)^. Although numerous factors may contribute to the development of SEA, potential causative factors should be distinguished from comorbid conditions because the risk factors must not only be shown to precede the disease but also be independently associated with its development. A list of potential causative factors is shown in Table 1.

**Table 1.** Risk Factors for SEA.

Source of infection	Causative condition
Recent spinal instrumentation	Diabetes mellitus
Epidural anesthesia	Abnormality of the vertebral column
Hemodialysis (Chronic renal failure)	Trauma of the spine
Consecutive bones or soft tissue infection	Intravenous drug use
Bacteremia from distant infection (Urinary tract, Respiratory tract, Abdomen, Endocarditis, Infected vascular access, and Dental abscess)	AIDS
	Malignancy
	Immunosuppressive therapy, Steroid use
	Alcoholism
Sepsis of unknown origin	Local spinal risk factors (degenerative disc disease, large osteophytes, hypertrophied facet joints)
Skin (Chronic nonhealing ulcers of the extremities)	

SEA; spinal epidural abscess

## 5. Diagnosis

### 5.1. Clinical manifestations

**Figure 2.**
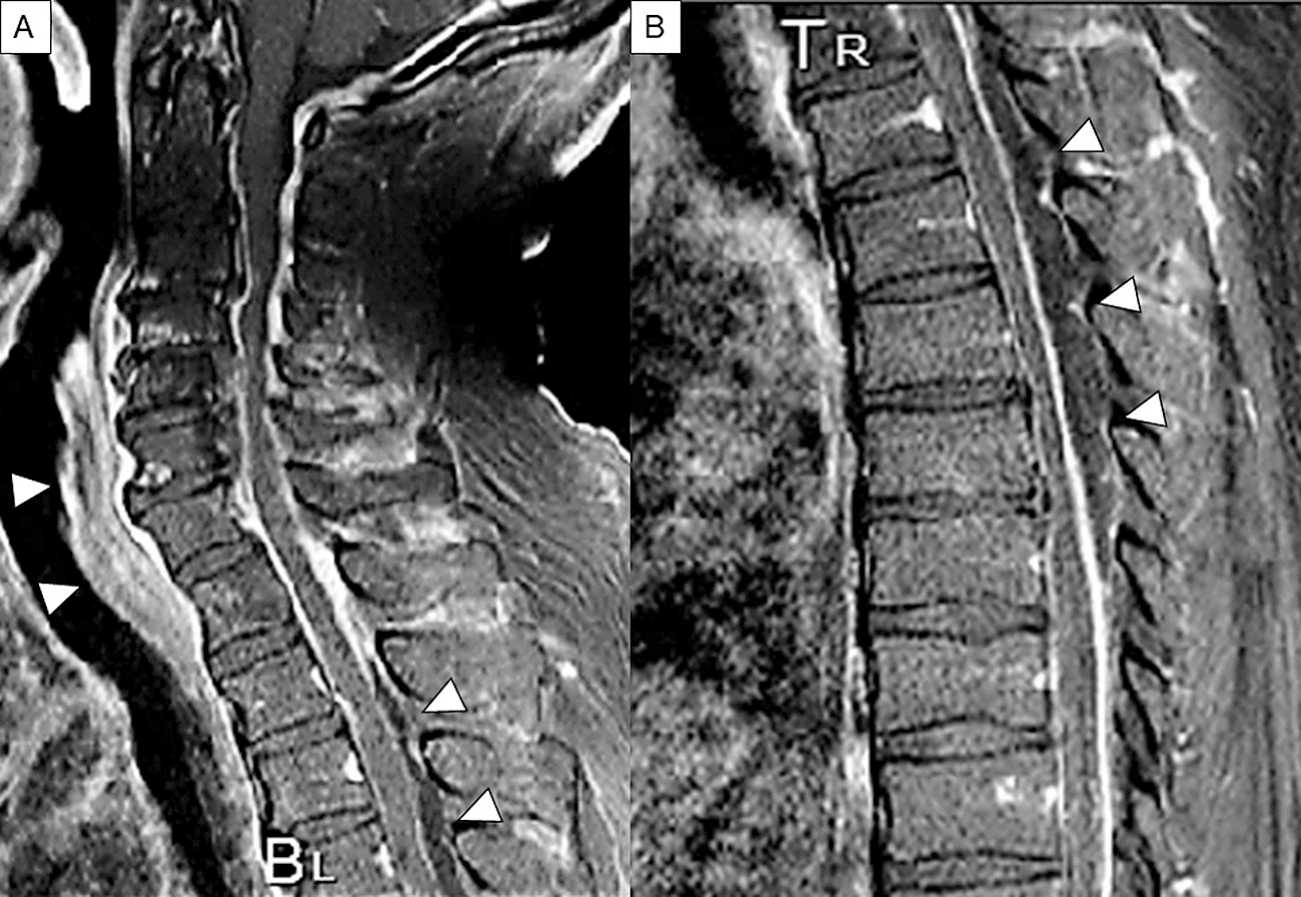
Sagittal Gd-enhanced T1-weighted MRI. Spinal epidural abscess is noted that a capsule-like rim whose edges are strongly enhanced and the content of abscess shows low intensity in the cervical (A) and thoracic (B) spine. The retro-pharyngeal abscess is also noted as enhanced mass (A). Arrowheads indicate the abscess.

The symptoms of SEA are characterized by rapid onset of back pain and fever. Fever, back pain, and neurological symptoms appear, which is described as a classical triad ^(5), (14), (15)^. Only 7.9% of cases have all three signs at the first assessment, and only 10% of cases present with all signs, even at the time of hospital admission ^(5)^. Only 0.8% of patients show all three signs but do not have SEA, and the classical triad is highly specific to SEA ^(5)^. The individual symptoms of the triad are not specific to SEA. Neurological manifestations, such as motor weakness, radiculopathy, and bladder and bowel dysfunction, have been reported in up to half of the cases ^(8)^. In instances of SEA in the cervical spine, neck pain and stiffness with lower-extremity weakness may be present. However, muscle weakness in the upper extremities can also be apparent, and some cases presented with tetraparesis (Figure 2A, B) ^(25), (26)^. Clinical progression is commonly classified into four stages: back pain in stage I, nerve root symptoms in stage II, muscle weakness and paresthesia in stage III, and complete paralysis in stage IV (Table 2) ^(27), (28)^. The duration of these symptoms and the degree of neurological deterioration should be determined because the severity of the neurological deficit could potentially evolve to complete paralysis within a few hours or days ^(29)^. Furthermore, the diagnosis of SEA is difficult to make because of its unpredictable course, so clinicians should frequently note neurological symptoms, as the transition from stages III to IV (i.e., irreversible paralysis) can occur promptly.

**Table 2.** Stages According to Clinical Severity in SEA.

Stage	Clinical manifestations
I	Back pain, fever, tenderness
II	Nerve root symptoms (radicular pain), nuchal rigidity/neck stiffness, decrease in tendon reflex
III	Muscle weakness, sensory abnormalities (hypesthesia, paresthesia, dysesthesia), bowel and bladder dysfunction
IV	Complete paralysis

Based on Heusner AP. Nontuberculous spinal epidural infections. N Engl J Med. 1948;239(23):845-54 ^(27)^ and Peterson JA, Paris P, Williams AC. Acute epidural abscess. Am J Emerg Med. 1987;5(4):287-90 ^(28)^.

### 5.2. Laboratory examinations

In SEA, inflammatory findings such as leukocytosis, elevated serum C-reactive protein (CRP) levels, and increased erythrocyte sedimentation rates (ESR) are noted. However, only leukocytosis occurs in about two-thirds of all patients, and ESR has higher sensitivities and is almost uniformly elevated in patients with SEA ^(5), (14), (15)^. Furthermore, the causative pathogen is isolated from blood culture in approximately 60% of patients ^(5), (30)^, but in all of them, blood cultures should be routinely analyzed for the appropriate selection of antibiotics when tissue cultures are not available or helpful. Additionally, direct aspiration of the abscess through lumbar puncture is useful for SEA diagnosis, but positive results are not always obtained. Given the increased risk of spreading the epidural infection through the lumbar puncture, resulting in cauda equina and conus medullaris syndrome, the diagnostic procedures that penetrate the subarachnoid space generally should be avoided until after SEA has been ruled out ^(14), (15)^.

### 5.3. Diagnostic imaging

**Figure 3.**
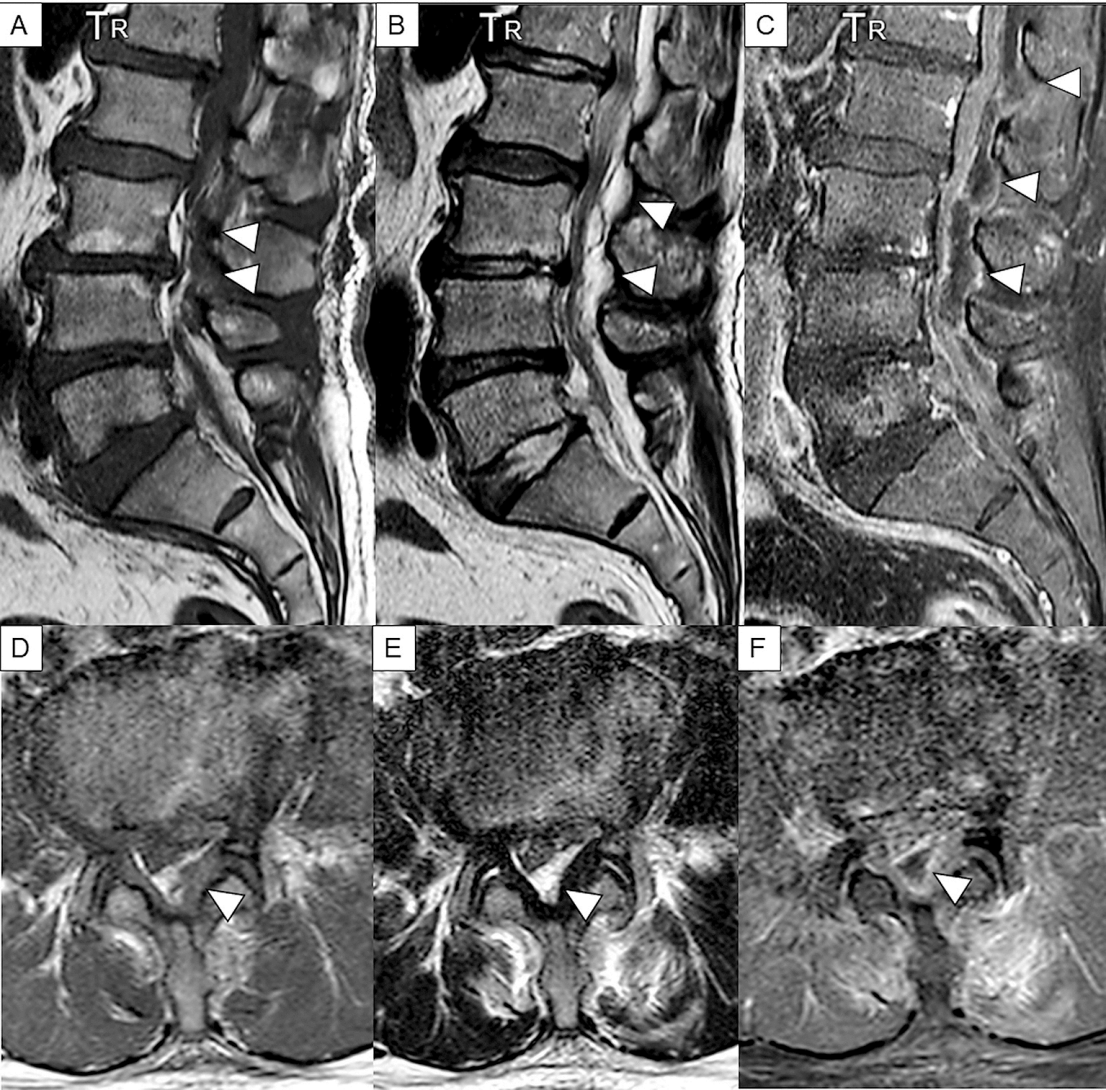
Sagittal and axial views of T1-weighted, T2-weighted, and Gd-enhanced T1-weighted MRI. Extensive epidural phlegmon is noted throughout the lumbar spine in the sagittal view; T1-weighted images show low signal intensity (A), T2-weighted images show high signal intensity (B), and Gd-enhanced MRI shows that a linear structure appears to be a thickened dural surface on the ventral side of the lamina. Acknowledge the occupying lesions inside and capsule-like rims whose edges are strongly enhanced. (C). The axial view at the level of L4 confirms that the dural tube is compressed by posterior SEA in each image (D–E). The enhancement is also observed in the L5 vertebral body and the paraspinal muscles, representing spondylitis and spread of inflammation into the surrounding muscles (C, F). Arrowheads indicate SEA.

**Figure 4.**
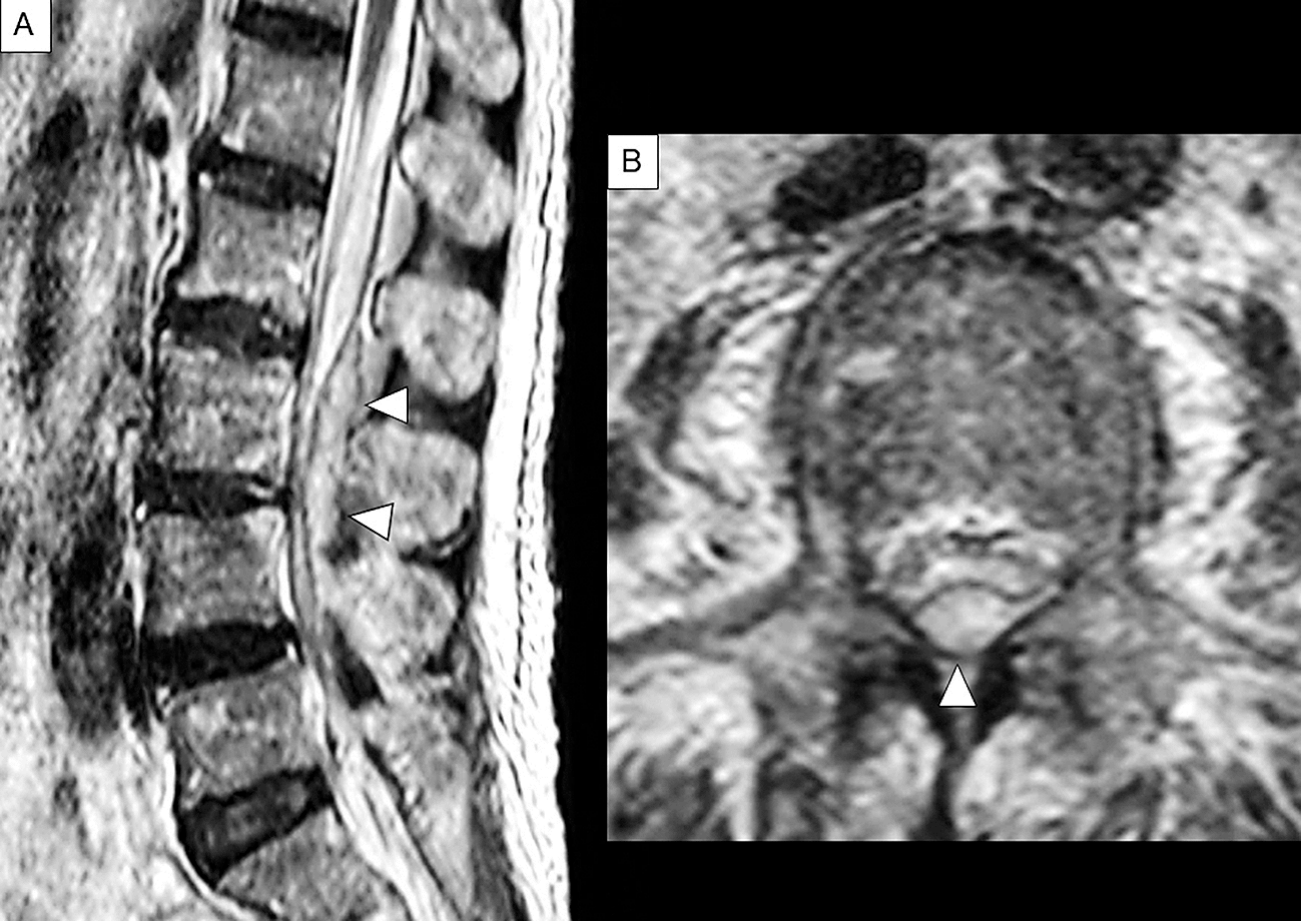
Sagittal and axial T2-weighted MRI in case 1. Extensive epidural phlegmon is noted throughout the lumbar spine in the sagittal view (A) and in the axial view (B). Content of abscess shows heterogeneous high intensity, accompanied by low intensity of capsule-like rim, which indicate granulation tissue (A). The axial view confirms that the dural tube is compressed posteriorly at the level of L3 (B). Arrowheads indicate SEA.

**Figure 5.**
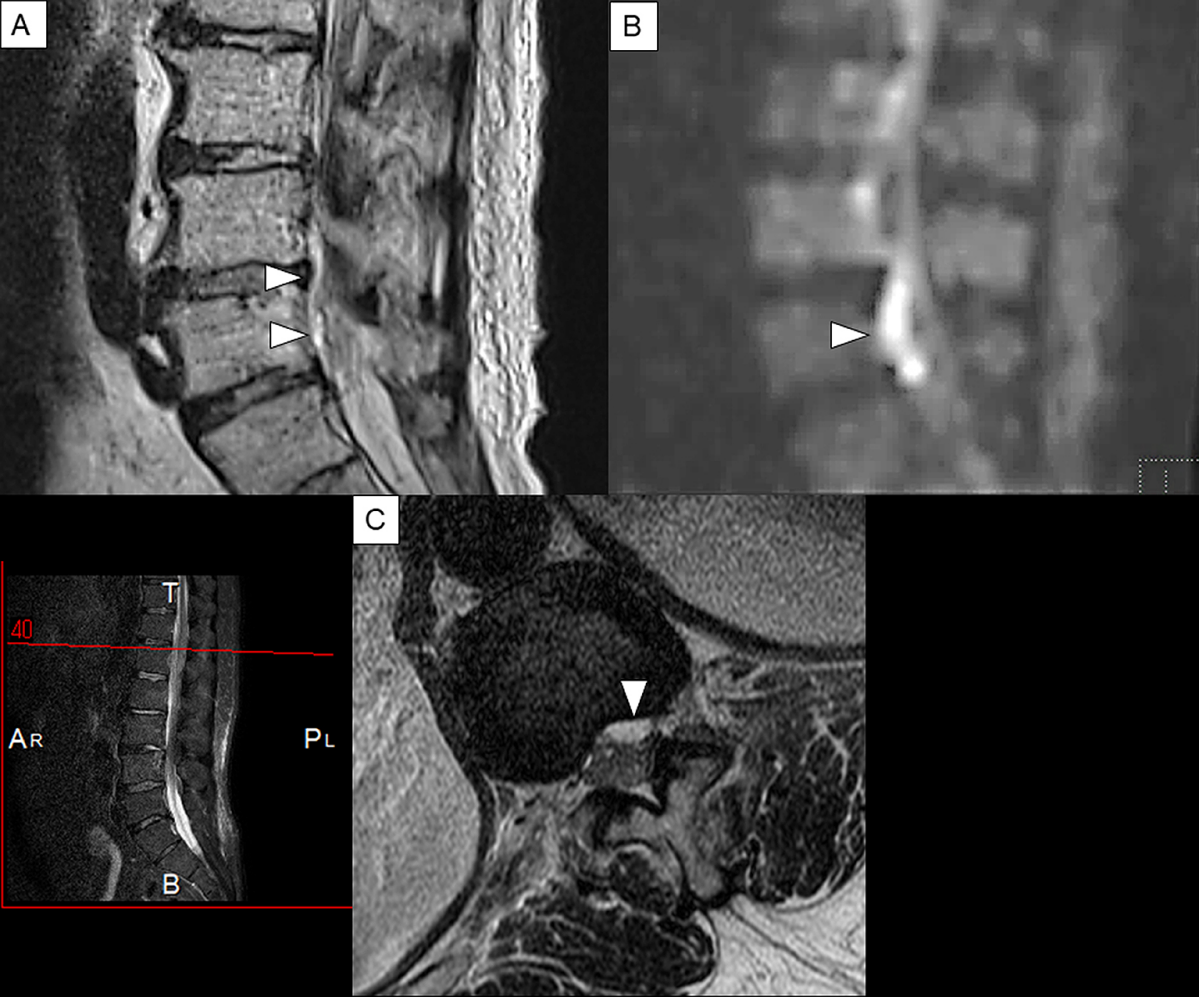
Sagittal T2-weighted and Gd-enhanced MRI of the lumbar spine in case2. (A): Sagittal view of T2-weighted MRI.(B): Sagittal view of diffusion-weighted image. (C): Axial view of T2-weighted MRI. A linear lesion that shows high intensity and appears to be an epidural abscess that is found on the dorsal side of the vertebral body (A). The same lesion shows a strong high signal area in the diffusion-weighted image (B). This finding is considered to reflect the decrease in diffusion of water molecules in the abscess lumen and is characteristic findings of abscess. The axial view confirms anterior SEA that shows high signal intensity on the dorsal side of the vertebral body at the level of L1 (C). Arrowheads indicate SEA.

MRI is a noninvasive, highly sensitive, and very specific imaging modality that can delineate the extent and location of the abscess. Its sensitivity to SEAs is 91% ^(22)^. The horizontal and sagittal tomographic images can show the extent of the lesions, thereby easily distinguishing SEA from spinal cord diseases such as spinal cord ischemia, acute transverse myelitis, and metastatic tumors ^(8)^. SEA tends to have low or intermediate intensity with the loss of cortical margins and continuity on T1-weighted images and high or intermediate intensity on T2-weighted ones. Liquid pus is related to an area of low signal intensity on T1-weighted images, but a rim of the abscess is enhanced after administration of Gadolinium (Gd)-contrast medium representing granulation tissue (Figure 3). In the case of phlegmonous inflammation, T2-weighted image shows high signal intensity where the epidural space is enlarged in accordance with active inflammation in the soft tissue. After Gd-enhancement, light or strong enhancement is clearly demonstrated, representing the extent of inflammation. After abscess formation, the puncta reservoir, showing high signal intensity on the T2-weighted image, is depicted as being surrounded by a capsule-like rim (Figure 4). This capsule-like structure at the border of the abscess cavity shows low signal intensity on the T2-weighted image (Figure 4), reflecting its abundant fibrous component. The surrounding rim of granulation tissue is strongly enhanced (Figure 3) ^(31)^. Furthermore, two patterns are seen on Gd-enhanced MRI according to the SEA stage. A homogenous enhancement of the abnormal area is recognized in the phlegmonous stage (at the early phase), which correlates with the granulomatous-thickened tissue with embedded microabscesses without a significant pus collection. A fluid abscess is surrounded by peripheral inflammation that is enhanced at the advanced phase ^(32)^. The inflammatory findings are sometimes seen in the vertebral body, intervertebral disk, and paraspinal muscles adjacent to SEA (Figure 3C, 3F). MRI can visualize the lesion from the earliest stage, and within 1 week of onset, it can confirm the presence of an abscess in the epidural space and the surrounding tissue. The diffusion-weighted image shows a strong signal, depicting the decrease in diffusion capacity of pus, and the apparent diffusion coefficient decreases. This feature effectively detects relatively small epidural abscesses (Figure 5). Although Gd-enhanced MRI is considered the most informative imaging modality for the detection of SEA, the extent of the infection can possibly be overestimated ^(33)^.

## 6. Management and Prognosis

The goal of treatment is to reduce the epidural abscess volume and eventually eliminate the abscess and causative bacteria through surgery and antibiotics. Surgery mainly involves laminectomy, degranulation, and pus drainage. Although the guidelines are not yet standardized for the surgical treatment selection, the reasons not to recommend surgery are as follows: (i) serious medical illness, (ii) absence of spinal cord compression symptoms, and iii) passage of more than three days from the onset to complete paralysis. As previously reported, emergency surgery (laminectomy and drainage) should be performed as soon as the diagnosis is established, if these three conditions do not apply ^(34)^. As venous congestion and thrombosis occur with the spread of the abscess, causing further neurological deteriorations, and once muscle weakness or motor nerve symptoms are noted, subsequent progression is rapid, and thus, careful follow-up and decision-making in the timing of surgical intervention are required. Generally, the postoperative improvement in the symptoms of paralysis is considerably poor in stage IV cases or patients with complete paralysis. In stage II cases, as previously reported, such improvement can be obtained in over 90% of cases if surgery is performed within 24 h ^(35)^. However, if surgery is performed 24–36 h after stage II, the prospect of recovery is likely diminished ^(8), (36)^. The progression from stages III to IV is often rapid, occurring within 24 h, especially if the abscess is extensive or if a serious underlying disease is noted. Some opinions then suggest immediate surgery at stages I and II without paralysis, and, in fact, most studies recommend that early surgery is beneficial to improve the outcome. However, the best exact timing for surgical intervention remains controversial ^(37), (38)^. Even if the symptoms are in stage I at the time of diagnosis, the rate of progression to subsequent stages is difficult to predict. Therefore, establishing a system to perform emergent surgery is desirable in preparation for the appearance of paralytic symptoms, i.e., when the progression to stage II or further is recognized, even when a conservative treatment plan is in place.

Antimicrobial agents are ideally selected for the causative organism identified from pus or blood cultures. Treatment should be started empirically without knowing the causative bacteria. Penicillin and first- or second-generation cephems, targeted *staphylococci*, should be administered, as these bacteria are most likely to cause SEA. If MRSA is suspected, vancomycin should be administered. Moreover, if UTI is suspected from the medical history, gram-negative bacilli are assumed to be present, and here, third- or fourth-generation cephem antibiotics are recommended ^(14), (15)^. Although the appropriate duration of antimicrobial therapy should be determined according to the responses of the clinical, laboratory, and radiological findings, the standard duration is from four to eight weeks ^(39)^. In addition, as previously reported, the relapse of SEA is reduced by administration of antibiotics for more than eight weeks ^(14), (15)^.

Recently, computed tomography (CT)-guided percutaneous needle aspiration may be a safe and minimally invasive procedure that can be regarded as a rational alternative to surgical intervention for the management of SEA in some patients, such patients without neurological deficits or those in poor medical condition ^(40)^.

The increase in MRI examinations has improved the opportunity to diagnose SEA at an extremely early stage. Therefore, in cases with minimal or no neurological signs at the time of diagnosis, good results can be possibly obtained through conservative treatment with antibiotics alone ^(14), (15), (26)^. In other words, patients diagnosed prior to the identification of neurological deficits can safely be treated only with antimicrobial therapy without decompression surgery, but those are accompanied by some risks of neurological deterioration. Careful neurological and imaging evaluation should be performed during conservative treatment. If the patient’s condition or neurological signs deteriorate and antimicrobial therapy fails, surgical intervention should be performed promptly ^(41)^. The timing of when to treat surgically remains difficult among patients with very mild neurological deficits.

Mortality due to SEA has decreased, but 5%–10% of patients died from sepsis, meningitis, and other complications ^(14), (15)^. SEA recurrence following therapy is rare. In a recent retrospective study, only about 3.6% (38 out of 1,053 patients with SEA) of cases recurred ^(42)^. In addition, although neurological sequelae have become more significant as mortality decreases, the extent of sequelae is strongly related to the degree of disability immediately at the start of treatment and the duration from the onset of paralysis to the start of treatment ^(14), (15)^.

## 7. Case Presentation

### Case 1

A 63-year-old woman presented with a one-week history of atraumatic lower back pain and fever. Given her past medical history of valvular heart disease, a bacterial infection such as infectious endocarditis was suspected. She had a bilateral lower-extremity weakness (grade 3/5) and was unable to walk. The deep tendon reflexes of both lower limbs were absent, and the pathological reflex was not detected. Her severity was in stage Ⅲ. Laboratory examination showed that white blood cell count and CRP level were 14,980/µL and 13.66 mg/dL, respectively, and MSSA was detected from cerebrospinal fluid culture. Sagittal T2-weighted MRI showed extensive epidural phlegmon throughout the lumbar spine with a capsule-like rim of low signal intensity along the periphery of the phlegmon. The axial views showed compression of the dural tube (Figure 4). Four days after admission, the patient was diagnosed with SEA and underwent posterior surgical decompression of the lumbar spine and drainage of the abscess. Intravenous antibiotics (ceftriaxone 2g IV q12h, vancomycin 0.5g IV q6h) was administered for 8 weeks. Postoperatively, a return of the strength (grade 4/5) of the iliopsoas and anterior tibialis muscles was noted bilaterally, but hypesthesia in both lower extremities persisted. She became able to walk with the assistance of a cane after rehabilitation. In this case, the neurological symptoms have not worsened despite the diagnostic delay, so surgical treatment could lead to neurological recovery. We initially suspected that the symptoms were possibly due to Guillain–Barré syndrome, so lumbar puncture was performed. As mentioned earlier, a lumbar puncture generally should be avoided after SEA has been ruled out. Once SEA is clinically suspected, a diagnostic procedure, particularly Gd-enhanced MRI, should be done urgently, and the treatment should be initiated promptly.

### Case 2

A 54-year old man with a history of lumbar spinal stenosis presented with a five-day history of severe low back pain, fever (temperature, 39°C), and vomiting. His limb weakness was not noted. His severity was stage Ⅰ. White blood cell count and CRP level were 7,850/μL and 28.2 mg/dL, respectively. MSSA was detected from blood culture. A linear lesion that appeared to be an epidural abscess was found along the dorsal side of the lumbar vertebral bodies on T2-weighted MRI (Figure 5). Subsequently, intravenous antibiotics (ceftriaxone 2g IV q12h, vancomycin 0.5g IV q6h) was administered for two weeks. However, paraplegia developed suddenly, and his severity abruptly became stage III. The patient then underwent emergent anterior lumbar discectomy and fusion. He became almost completely paraplegic (Stage Ⅳ) and showed no improvement after rehabilitation. He could not void and was discharged with a urinary catheter. In this case, although he was diagnosed with SEA in the early stage and treated with antibiotics alone, the timing of the operation may have been delayed. If the symptoms are in stage I at the time of diagnosis like in this case, the progression to subsequent stages is difficult to predict. In addition, the deterioration of neurological deficits in SEA can be sudden. Thus, clinicians should be aware of these features to prevent delay in surgical intervention.

## 8. Strategies for Early Diagnosis and Appropriate Management

Despite advances in diagnostic imaging, antibiotic therapies, and surgical techniques, almost half of SEA survivors have neurological sequelae, including 15% of those left with paresis or complete paralysis ^(43)^. The appropriate diagnosis was delayed in approximately 75% of patients who were eventually diagnosed with SEA. Muscle weakness remained in 45% of these patients. On the other hand, in patients who had not experienced a diagnosticdelay, muscle weakness was reported to have remained in only 13% of cases ^(5), (44)^.

Patients who do not improve with nonoperative management alone and experience delayed surgery have a poor outcome, with rates of approximately 30%–40%, as previously reported ^(45), (46)^. Immediate surgery with antibiotics is suggested to improve neurological outcome compared with delayed surgery after the failure of nonoperative management ^(45), (47)^. Early surgery with intravenous antibiotics might be the best choice for many patients, particularly those with progressive neurologic deficits.

However, there are some reports documenting recovery in SEA patients who were treated with antibiotics alone, avoiding the risk of surgery. Some previous studies suggested that SEA should be mainly treated conservatively in patients with no or minimal neurological symptom ^(48), (49)^. Medical plus surgery versus antibiotics alone remains controversial. There are no randomized trials of conservative versus surgical management of SEA at present, and it will be difficult to do such research in the future. Therefore, we wish to identify which patients are likely to fail nonoperative management. Predictive factors for failure of treatment with antibiotics alone have been reported, which include extremely elevated CRP, leukocytosis, bacteremia, age older than 65, and MRSA as the pathogen ^(39), (45), (50)^. In the most recent study, risk or predictive factors for failure of nonoperative management are introduced in which six independent predictors are listed: motor deficit at presentation, pathologic or compression vertebral fracture in the affected levels, active malignancy, diabetes mellitus, sensory change, and dorsal location of abscess (Table 3) ^(51)^. These findings may provide a useful tool for clinicians in weighing the risks and benefits of initial nonoperative management.

**Table 3.** Predictive Factors for Failure of Nonoperative Management.

Diabetes mellitus
Elevated CRP (CRP level >115 mg/L (11.5 mg/dL)
Leukocytosis (white blood cell count >12,500 cells/L)
Bacteremia
Physician-documented decline in motor status while on intravenous antibiotics
Age older than 65
MRSA
Severe neurological involvement
Motor deficit at presentation
Pathologic or compression vertebral fracture in the affected levels
Active malignancy
Sensory change
Dorsal location of abscess
Primarily radiographic evidence of disease progression
Progression of bony deformities in the spine

CRP; C-reactive protein, MRSA; methicillin‐resistant Staphylococcus aureus. Based on Shah AA, Ogink PT, Nelson SB, et al. Nonoperative management of spinal epidural abscess: Development of a predictive algorithm for failure. J Bone Joint Surg Am. 2018;100(7):546-55 ^(51)^.

Early diagnosis and appropriate management are keys to a good outcome. The clinical signs, duration of symptoms, and rate of the deterioration of neurological findings vary among patients with SEA. As mentioned, in the early stages of the disease, the so-called classical triad (back pain, fever, and neurological deficits) seldom appear simultaneously. Severe localized low back pain is the most frequently reported symptom and can be the trigger for more specific clinical testing leading to correct diagnosis. When examining a patient, a complete neurological examination, including sensory and motor functions, reflexes, and gait, must be performed. In addition, cases with suspected or identified SEA require frequent careful neurological examinations. Patients who are highly suspicious of SEA with neurological deficit, severe focal back pain, unexplained fever, and elevated CRP or ESR should undergo emergent MRI. Every effort should be made to prevent delay in diagnosis. A recent protocol-based approach to emergent MRI to diagnose SEA includes five risk factors (intravenous drug use, indwelling vascular catheter, spinal procedure or infection within six months, antimicrobial treatment within 30 days, and infectious focus elsewhere), resulted in an immediate identification and reduction of time required in the identification of patients with suspected SEA. Thus, the use of the protocol seemingly facilitates the early diagnosis of SEA ^(52)^.

**Figure 6.**
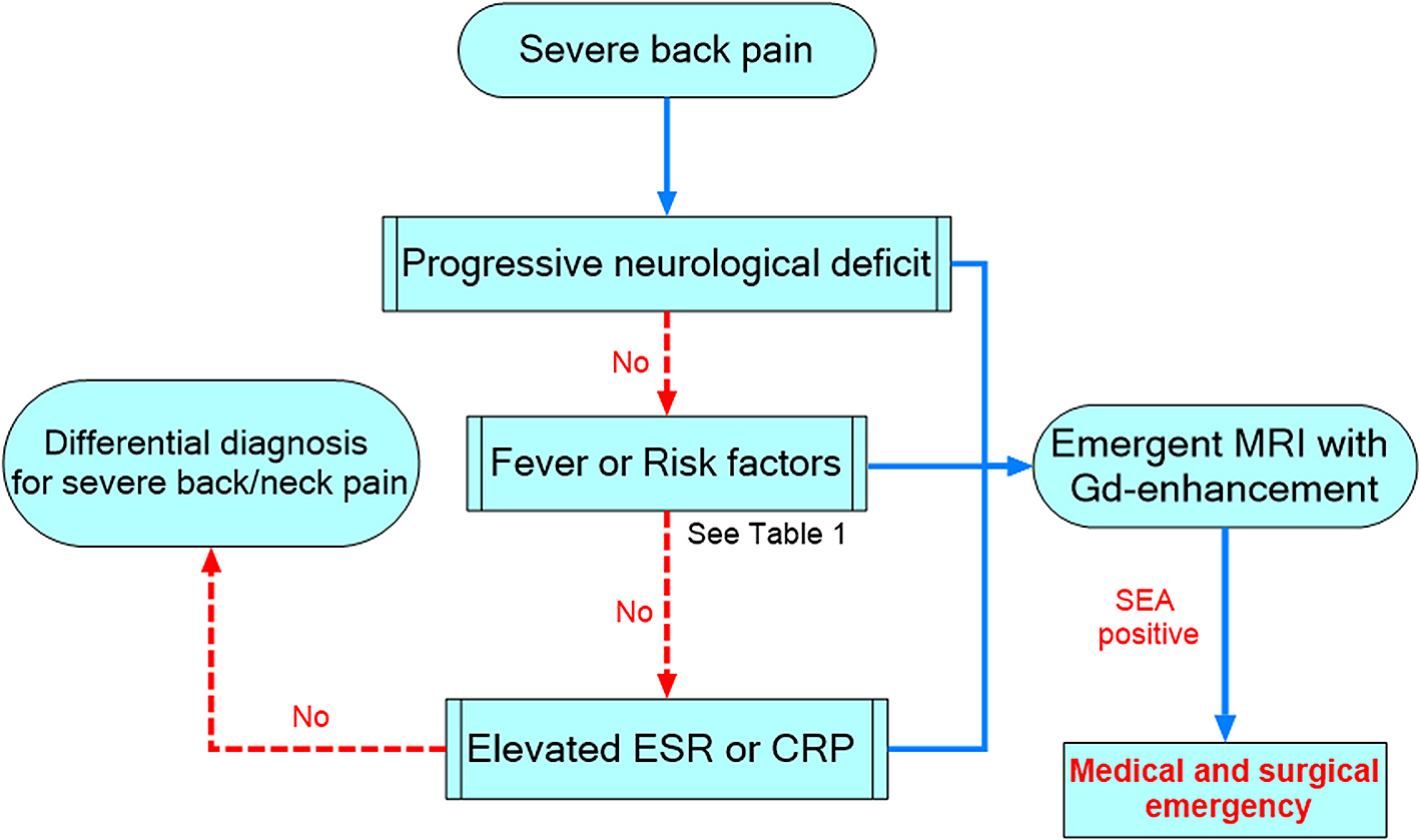
Algorithm for early diagnosis of SEA in patients with severe back pain, using the assessment of risk factors and testing of ESR or CRP. SEA, spinal epidural abscess; CRP, C-reactive protein; ESR, erythrocyte sedimentation rate.

By summarizing the aforementioned and referring recent recommendations ^(52), (53), (54)^, we proposed a simple algorithm for the early diagnosis of SEA (Figure 6). At first, severe back pain is an essential symptom to make clinicians suspect SEA because most patients complain of severe localized back pain. Next, careful neurological examination and assessment of risk factors or predisposing conditions and laboratory examinations, including CRP or ESR, should be done. Because elevated ESR or CRP level was found in most cases of SEA, emergent MRI should be considered for patients who complain of severe back pain with elevated inflammatory markers, even with unclear risk factors. This algorithm may bring us one step closer to the standardization of the potential strategies for the early diagnosis of SEA and would help clinicians evaluate individual features while considering the differential diagnosis.

## 9. Conclusions

SEA can lead to potentially devastating and even death, and continues to challenge clinicians in its diagnosis and management. The most significant prognostic factor for a favorable outcome in patients with SEA is early diagnosis and appropriate treatment before the emergence of neurological symptoms. Every effort should be made to shorten the diagnostic delay. Gd-enhanced MRI is the most beneficial imaging modality for establishing the correct diagnosis of SEA. Patients with SEA presenting neurological deficits should undergo immediate surgical decompression. Patients with no neurological deficit with a known causative pathogen can be treated with antibiotics alone. If the patient’s condition or neurological symptoms deteriorate and antimicrobial therapy alone fails, surgical intervention should be performed promptly. Efforts for establishing clear indications for surgical decompression in SEA with no or mild neurological deficits are underway.

## Article Information

### Conflicts of Interest

None

### Acknowledgement

The authors would like to thank Enago (www.enago.jp) for the English language review.

### Author Contributions

All authors contributed equally to this work.

### Approval by Institutional Review Board (IRB)

This manuscript is a review article and does not need approval by IRB.
